# Urban–rural and comorbidity-related differences in vaccination attitudes and self-reported adverse events following immunisation in Poland: a cross-sectional primary care study

**DOI:** 10.3389/fpubh.2026.1871915

**Published:** 2026-09-18

**Authors:** Dorota Stefanicka-Wojtas, Agnieszka Rusiecka, Tomasz Hikawczuk, Aneta Soll-Morka, Maria Kasprzyk-Smardz, Monika Zadka, Norbert Zachara, Jarosław Zachara, Donata Kurpas

**Affiliations:** 1 Division of Scientific Research Methodology, Department of Nursing, Faculty of Nursing and Midwifery, Wroclaw Medical University, Wroclaw, Poland; 2 Statistical Analysis Centre, Wroclaw Medical University, Wroclaw, Poland; 3 Medra Kasprzyk-Smardz & Partners, Kępno, Poland; 4 Non-Public Healthcare Centre “Przychodnia Nowy Dwór”, Wroclaw, Poland; 5 Non-Public Healthcare Centre “ReMed”, Borzęcin, Poland

**Keywords:** adverse events following immunisation, comorbidity, urban–rural differences, vaccination, vaccine hesitancy

## Abstract

**Background:**

Vaccination attitudes and uptake are shaped by sociodemographic context, geography, and health status. How comorbidities and prior infection experiences interact with place of residence to influence vaccine decision-making remains insufficiently characterized in Polish primary care settings.

**Objective:**

To compare vaccination attitudes, self-reported adverse events following immunisation (AEFI), and determinants of vaccination across large-city, small-city, and rural primary care populations in Poland, with particular attention to comorbidities and prior infectious disease experiences.

**Methods:**

We conducted a cross-sectional study using questionnaire data linked to primary care records in three primary healthcare centres representing a large city, a small-to-medium city, and a rural setting. Group differences were assessed with contingency tables and Pearson’s chi-square tests (two-sided alpha 0.05). Multivariable logistic regression modeled uptake of influenza, COVID-19, and pertussis vaccination using comorbid respiratory, circulatory, and metabolic conditions and prior infection history as predictors.

**Results:**

Vaccination coverage differed markedly by setting (*p* < 0.001), reaching 100% in the rural centre compared with 73–76% in urban centres. Self-reported AEFIs were least frequent among rural participants (7.1%) and most frequent among large-city participants (20.0%) (*p* < 0.001). Perceived information availability and quality, as well as trust in the healthcare system, varied across sites (all *p* < 0.001). In adjusted models, prior infection history and respiratory comorbidity were associated with higher odds of influenza vaccination (OR = 11.0; *p* < 0.001 and OR=3.9; *p* = 0.001, respectively) and COVID-19 vaccination (OR = 3.6; *p* < 0.001 and OR = 2.8; *p* = 0.010). Circulatory and metabolic conditions were not associated with influenza or COVID-19 vaccination (*p* > 0.05) but were inversely associated with pertussis vaccination in this sample (both *p* < 0.01).

**Conclusion:**

Vaccination attitudes, uptake, and self-reported AEFIs differ substantially between urban and rural primary care settings in Poland. Respiratory comorbidity and prior infection experiences consistently predict vaccine uptake, underscoring the need for geographically tailored communication strategies and risk framing that reflects local context and chronic disease burden.

## 1 Introduction

Vaccination remains one of the most effective public health interventions, yet substantial disparities in vaccine uptake persist across social and geographic strata (1–4). Vaccine hesitancy is now recognized as a multifactorial and context-dependent phenomenon shaped by confidence, complacency, and constraints, as well as broader psychosocial, cultural, and political determinants (2–4). Importantly, these determinants rarely operate in isolation. Instead, they are embedded within local health systems, community norms, and information environments that shape how risks and benefits are perceived.

Urban and rural populations differ systematically in healthcare accessibility, exposure to health information, and institutional trust, all of which influence vaccination decision-making (4–7). In Europe, and particularly in Central and Eastern Europe, vaccine hesitancy reflects not only individual-level beliefs but also structural and historical factors, including trust in public institutions, perceived transparency of vaccination policy, and experiences during the COVID-19 pandemic (8–10). These contextual elements may interact with local social dynamics, contributing to marked within-country variability in vaccine uptake.

Comorbidities such as respiratory, cardiovascular, and metabolic disorders are established clinical indications for vaccination and may influence decision-making through perceived vulnerability and physician recommendation (11, 12). However, the relationship between chronic disease burden and vaccination attitudes remains inconsistently documented. Some studies suggest that individuals with chronic conditions are more likely to vaccinate due to higher perceived risk and anticipated protective benefits (11–13). In contrast, others report no association or even increased hesitancy related to safety concerns, fear of adverse events, or apprehension regarding interactions with ongoing treatment (3, 8, 14). Whether specific comorbidity profiles translate into greater vaccine uptake depends on both clinical communication and local informational context.

Exposure to infectious disease, either through personal experience or within the household, further shapes risk perception and preventive behavior (4, 6, 12, 14). Rural residents often report closer relationships with primary care physicians and higher trust in local healthcare providers, which may reinforce vaccine acceptance (5, 6, 10, 15). At the same time, rural communities may face logistical constraints, including geographic distance and limited service availability. Urban populations, in contrast, typically have greater access to services but are also exposed to more heterogeneous information sources, including misinformation and vaccine-critical narratives, which may amplify concerns regarding safety and adverse events (1, 2, 4, 14).

Despite growing literature on vaccine hesitancy, few studies have examined how geographical context, comorbidity burden, and prior infection history jointly shape vaccination attitudes, uptake, and adverse event reporting within Central and Eastern European primary care settings. Poland represents a particularly informative context. Although childhood vaccination coverage remains high, adult vaccination and booster uptake show substantial regional and sociodemographic variability (10, 13, 16–20). Public debate during the COVID-19 pandemic highlighted divisions in trust, safety, and confidence in national vaccination policy, especially regarding novel vaccines and evolving recommendations (10, 13, 16, 17). However, empirical evidence integrating clinical vulnerability and urban–rural context within routine primary care populations remains limited. A better understanding of these intersecting determinants is essential for designing context-sensitive public health strategies, strengthening clinician-patient communication, and addressing disparities in vaccine confidence. Poland maintains high coverage rates for mandatory childhood vaccinations through a long-established national immunisation program. However, increasing public debate over vaccine safety and growing vaccine hesitancy among some parents have raised concerns about future vaccination coverage. Because caregivers responsible for childhood immunisation decisions were among the study groups, understanding factors associated with vaccination attitudes across both adult and pediatric contexts is particularly relevant.

This study aimed to examine urban–rural differences in vaccination uptake, vaccination attitudes, and self-reported adverse events following immunisation in Polish primary care populations, and to determine whether respiratory, cardiovascular, and metabolic comorbidities, as well as prior infectious disease history, independently predict vaccination decisions. We hypothesized that rural populations would demonstrate higher vaccination uptake and lower reporting of adverse events compared with urban populations, and that respiratory comorbidity and prior infection experience would be positively associated with vaccination.

## 2 Materials and methods

### 2.1 Study design and settings

This cross-sectional study integrated newly collected questionnaire data with selected information from primary care medical records concerning vaccination practices. The survey targeted three groups: individuals who accepted vaccination, individuals who declined vaccination, and caregivers responsible for decisions regarding childhood immunisation. The questionnaire assessed attitudes and experiences related to vaccination in general rather than focusing on a single vaccine type. Information was collected on the uptake of various vaccines administered across the lifespan, including childhood vaccinations and selected adult vaccines, such as influenza, COVID-19, and pertussis. Specific vaccine types were analyzed separately when sufficient numbers were available for statistical modeling.

The study was conducted in three purposively selected Primary Healthcare Centres (PHCs) representing distinct contextual environments: a large urban centre, a medium-sized urban centre, and a rural centre. Linking questionnaire responses to medical records required prior bioethical approval and coordinated data access procedures at each participating PHC. The participating primary healthcare centres were engaged through a project-specific procurement procedure. Site selection was determined primarily by administrative, legal, and operational requirements, including willingness to participate and the ability to provide access to the required data. The centres that ultimately participated represented three different healthcare contexts: a large urban centre, a medium-sized urban centre, and a rural centre. This configuration enabled comparison of vaccination attitudes and behaviors across contrasting primary care environments; however, the study was not designed to provide nationally representative estimates.

Participants were recruited consecutively during routine visits at the participating primary healthcare centres. Recruitment was conducted face-to-face by healthcare personnel. All eligible adult patients and caregivers responsible for childhood vaccination decisions attending the participating centres during the study period were invited to participate. Inclusion criteria comprised age ≥18 years or legal responsibility for vaccination decisions regarding a child, attendance at one of the participating centres, and provision of consent. The sole exclusion criterion was failure to provide consent to participate in the study.

Data collection was completed across all sites, yielding 1,224 records (PHC 1: *n* = 408; PHC 2: *n* = 415; PHC 3: *n* = 401). The consolidated dataset includes questionnaire responses and selected variables extracted from medical registers, encompassing vaccinated and unvaccinated adults and children, as well as healthcare personnel. Each participant was assigned a unique study identifier to ensure accurate linkage between self-reported data and corresponding medical records. Only individuals who provided consent for data linkage were included in the integrated analytical dataset (Figure 1).

**Figure 1:**
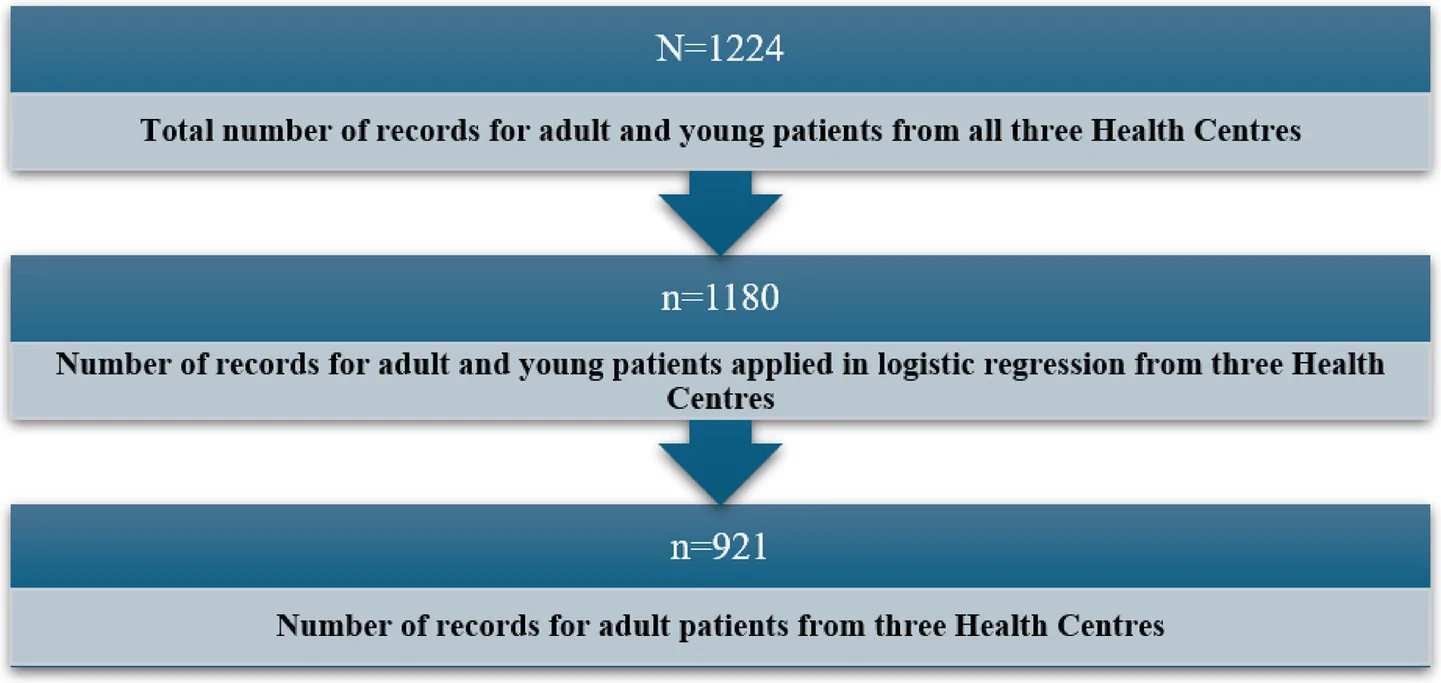
Scheme of analytic samples for logistic regression analysis and contingency table comparisons.

The resulting dataset enabled:

1. Identification of sociodemographic and clinical characteristics associated with vaccination status and related health behaviors;
2. Assessment of factors influencing vaccination decisions among adults and parents;
3. Evaluation of trust in the healthcare system and attitudes toward mandatory vaccination;
4. Analysis of concerns regarding adverse events following immunisation and their determinants.

For the analytic sample reported in this manuscript in the form of contingency tables, data from 310 participants in the large urban PHC, 372 in the medium-sized urban PHC, and 239 in the rural PHC were included. Adult participant data were compiled in a structured database format for statistical analysis (*n* = 921). Additionally, in logistic regression analysis, data for 1,180 patients (adults and children/caregivers) from 1,224 were taken into consideration (after considering missing data for any of the independent variables).

The primary study endpoint was vaccination uptake, defined as vaccination status (vaccinated versus unvaccinated). Secondary outcomes included attitudes toward vaccination, trust in the healthcare system, perceived quality and availability of vaccination-related information, social influences on vaccination decisions, attitudes toward mandatory vaccination, and self-reported adverse events following immunisation (AEFI). Exploratory analyses examined associations between vaccination uptake and selected clinical factors, including prior history of infectious disease and respiratory, circulatory, and metabolic comorbidities.

### 2.2 Data collection and verification

All collected data were reviewed for completeness and internal consistency before analysis. Questionnaire responses from each participating health centre were cross-checked against the submitted source documents to identify missing values, inconsistencies, and data-entry errors.

Data verification and initial cleaning were conducted using Microsoft Excel 2019 (Microsoft Corporation, Redmond, WA, USA). This process ensured accurate record linkage, consistent variable coding, and completeness of key analytical variables before transfer to the statistical analysis software.

### 2.3 Statistical analysis

The data were analyzed in Statistica 13.3 software (TIBCO Software Inc., Palo Alto, CA, USA). Contingency tables were created for the variables being compared, using percentage frequencies for adult patients at each health centre. In the next step, the difference between observed and expected frequencies for each contingency table was evaluated using Pearson’s χ^2^ test. Statistical significance was considered at *p* < 0.05.

#### 2.3.1 Logistic regression model

Additionally, logistic regression was used to create a model of vaccination rates for the diseases included in the personal questionnaires for adult and pediatric patients based on data from three Health Centres (*n* = 1,180). Of the 1,224 individuals, those with missing data on any variable were excluded from the logistic regression model. Different logistic regression models were analyzed for the three most common diseases in the collected dataset: flu, SARS-CoV-2, and pertussis, as dichotomous variables. For each model, independent variables were analyzed, such as prior illness and the presence of respiratory, circulatory, and metabolic diseases. Answers were coded as 0 (no) and 1 (yes).

The impact of all independent variables on the dichotomous vaccination status variable was assessed using the likelihood ratio χ^2^ test (*p* < 0.05), and the effect of each independent variable was evaluated separately using the Wald χ^2^ test (*p* < 0.05). Odds Ratios (OR) and 95% Confidence Intervals (CI) were also determined for each independent variable and intercept.

#### 2.3.2 Parameters of the logistic regression model

To assess multicollinearity among the independent variables, the variance inflation factor (VIF) was calculated. To assess the extent to which the independent variables predict the dichotomous variable, the Nagelkerke R^2^ coefficient was calculated. To compare individual models, two information criteria were used: the Akaike information criterion (AIC) and the Bayesian information criterion (BIC). The Area Under the Curve (AUC) value was used to determine the model’s predictive ability to distinguish between willingness to vaccinate and not to vaccinate.

## 3 Results

### 3.1 Study population and baseline characteristics

Table 1 presents administrative data for the study participants. In the case of vaccinations, a significant difference between observed and expected values (*p* < 0.05) was found in the number of vaccinated and unvaccinated individuals within individual centres.

**Table 1:** Administrative data of patients participating in the study.

Item	Health centre			χ^2^ value	*p*-value*
	Large city (*n* = 310)	Small city (*n* = 372)	Village (*n* = 239)		
Vaccination (df = 2)					
Yes	235 (75.8%)	272 (73.1%)	239 (100%)	76.507	<0.001
No	75 (24.2%)	100 (26.9%)	0 (0%)		
Sex (df = 2)					
Female	176 (56.8%)	214 (57.5%)	154 (64.4%)	3.888	0.143
Male	134 (43.2%)	158 (42.5%)	85 (35.6%)		
Age (df = 6)					
18–38	89 (28.7%)	62 (16.7%)	58 (24.3%)	31.238	<0.001
39–59	134 (43.2%)	141 (37.9%)	91 (38.1%)		
60–80	76 (24.5%)	159 (42.7%)	87 (36.4%)		
>81	11 (3.5%)	10 (2.7%)	3 (1.3%)		
Education (df = 6)					
Primary	28 (9.0%)	26 (7.0%)	23 (9.6%)	75.425	<0.001
Secondary	145 (46.8%)	200 (53.8%)	136 (56.9%)		
Higher	128 (41.3%)	90 (24.2%)	35 (14.6%)		
Other	9 (2.9%)	56 (15.1%)	45 (18.8%)		
Profession (df = 8)					
Not related to medicine	294 (94.8%)	347 (93.3%)	229 (95.8%)	7.412	0.493
Nurse	12 (3.9%)	18 (4.8%)	7 (2.9%)		
Doctor	1 (0.3%)	6 (1.6%)	1 (0.4%)		
Pharmaceutical technician	2 (0.6%)	1 (0.3%)	1 (0.4%)		
Emergency medical technician	1 (0.3%)	0 (0.0%)	1 (0.4%)		
Income (df = 14)					
up to 2000 zł	2 (0.6%)	11 (3.0%)	4 (1.7%)	182.243	<0.001
2001–3,000 zł	26 (8.4%)	72 (19.4%)	15 (6.3%)		
4,001–5,000 zł	55 (17.7%)	67 (18.0%)	16 (6.7%)		
5,001–6,000 zł	39 (12.6%)	30 (8.1%)	7 (2.9%)		
6,001–8,000 zł	50 (16.1%)	23 (6.2%)	7 (2.9%)		
8,001–10,000 zł	26 (8.4%)	20 (5.4%)	9 (3.8%)		
more than 10,000 zł	29 (9.4%)	6 (1.6%)	12 (5.0%)		
I prefer not to say	83 (26.8%)	143 (38.4%)	169 (70.7%)		
Place of residence (df = 6)					
Village	21 (6.8%)	125 (33.6%)	235 (98.3%)	1106.73	<0.001
City < 50 k inhabitants	6 (1.9%)	221 (59.4%)	3 (1.3%)		
City 50–200 k inhabitants	11 (3.5%)	21 (5.6%)	1 (0.4%)		
City > 200 k inhabitants	272 (87.7%)	5 (1.3%)	0 (0.0%)		
Religion (df = 12)					
Catholicism	244 (80.5%)	356 (95.7%)	232 (97.1%)	59.644	<0.001
Orthodoxy	20 (6.6%)	7 (1.9%)	1 (0.4%)		
Protestantism	2 (0.7%)	0 (0.0%)	0 (0.0%)		
Buddhism	1 (0.3%)	0 (0.0%)	0 (0.0%)		
Islam	2 (0.7%)	0 (0.0%)	0 (0.0%)		
Jehova’s Witnesses	3 (1.0%)	0 (0.0%)	0 (0.0%)		
Other	31 (10.2%)	9 (2.4%)	6 (2.5%)		

^*^p-value statistically significant at *p* < 0.05.

### 3.2 Vaccination coverage by setting

The rural centre had complete vaccination coverage, while in the urban centres it ranged from 73.1 to 75.8%. There was no significant gender difference between the centres, ranging from 56.8% in the large city health centre to 64.4% in the rural centre (*p* > 0.05). However, statistically significant differences were observed in terms of age range (*p* < 0.01). The largest proportion of patients in all centres was aged 39–59 (37.9–43.2%), followed by older adults aged 60–80 (24.5–42.7%). Statistically significant differences between observed and expected values (*p* < 0.01) were also found in educational level. The largest percentage in the study was among individuals with secondary education (ranging from 46.8% in the large city to 56.9% in the rural area). The large city health centre also had a higher proportion of people with higher education (41.3%) than the rural centre (14.6%). A higher percentage of patients in the rural centre and small-town centre also declined to disclose their education (18.8 and 15.1%, respectively). No significant differences in values were found between medical and non-medical professions across the centres (*p* > 0.05). There was a difference in values across residents’ income levels (*p* < 0.01), with a higher proportion of higher-income residents observed in the larger city. However, as population size decreased, the percentage of people in smaller towns and rural areas who chose not to disclose their monthly income increased (from 38.4% to as high as 70.7%). The centre’s location also differentiated patients by place of residence (*p* < 0.01). In the large city, 87.7% of patients were residents, while in the small town, 59.4% were from the city and 33.6% from surrounding villages. In the rural centre, rural residents constituted 98.3%. Significant difference (*p* < 0.01) was also found in religious denomination. The predominant denomination was Catholicism, with proportions ranging from 97.1 to 80.5% in rural and urban centres, respectively. The urban centre also had a higher proportion of patients of the Orthodox faith (6.6%) and a 10.2% share who selected “other” in the survey.

### 3.3 Perceived information environment and trust in the healthcare system

Table 2 includes information on vaccination availability and quality, as well as the level of trust in healthcare. Significant difference in responses (*p* < 0.01) was found across health centres. The most common survey responses to the question about access to information on vaccinations were “medium” and “good,” with 35.8% of responses in both categories at the large city health centre. At the small-town health centre, “good” responses were more frequent than “medium” responses (45.7 and 22.6%, respectively). In rural areas, “medium” responses were more frequent than “good” responses (49.4% vs. 40.2%). Similar differentiation was observed for information quality (*p* < 0.01), and the values obtained were also statistically significant when compared with expected values. Statistical differences were also observed for the question regarding trust in healthcare (*p* < 0.01). Among all the centres analyzed, the “medium” response was most common, with the lowest share in small towns (48.4%) and the highest in rural areas (61.9%). The range of good responses was 23.2 to 30.5% for the centres located in a large city and in the countryside, respectively.

**Table 2:** Opinions about the health care system and vaccinations.

Item	Health centre			χ^2^ value	*p*-value*
	Large city (*n* = 310)	Small city (*n* = 372)	Village (*n* = 239)		
How do you assess the availability of vaccination information? (df = 10)					
I do not have an opinion	15 (4.8%)	43 (11.6%)	6 (2.5%)	91.796	<0.001
Very weak	7 (2.3%)	15 (4.0%)	1 (0.4%)		
Weak	46 (14.8%)	25 (6.7%)	9 (3.8%)		
Medium	111 (35.8%)	84 (22.6%)	118 (49.4%)		
Good	111 (35.8%)	170 (45.7%)	96 (40.2%)		
Very Good	20 (6.5%)	35 (9.4%)	9 (3.8%)		
How do you rate the quality of information on vaccinations? (df = 10)					
I do not have an opinion	13 (4.2%)	39 (10.5%)	6 (2.5%)	83.568	<0.001
Very weak	9 (2.9%)	13 (3.5%)	1 (0.4%)		
Weak	43 (13.9%)	36 (9.7%)	9 (3.8%)		
Medium	111 (35.8%)	84 (22.6%)	121 (50.6%)		
Good	112 (36.1%)	165 (44.4%)	95 (39.7%)		
Very Good	22 (7.1%)	35 (9.4%)	7 (2.9%)		
How do you assess confidence in the healthcare system? (df = 10)					
I do not have an opinion	7 (2.3%)	22 (5.9%)	0 (0.0%)	49.625	<0.001
Very weak	15 (4.8%)	19 (5.1%)	3 (1.3%)		
Weak	39 (12.6%)	32 (8.6%)	9 (3.8%)		
Medium	165 (53.2%)	180 (48.4%)	148 (61.9%)		
Good	72 (23.2%)	94 (25.3%)	73 (30.5%)		
Very Good	12 (3.9%)	25 (6.7%)	6 (2.5%)		

*p-value statistically significant at *p* < 0.05.

### 3.4 Self-reported AEFI among participants

Table 3 presents the results of the comparison of AEFI incidence across individual health centres. For most of the variables compared, significant differences in AEFI frequency were found between individual centres when observed data were compared with expected values (*p* < 0.05). A significantly higher incidence of fever (*p* < 0.01) was observed in the large city compared with the rural health centre, ranging from 16.1 to 5.4%. The same significant (*p* < 0.01) incidence was observed for swelling at the vaccination site, ranging from 11.9 to 1.3% in the large city and the rural health centre, respectively. In the case of enlarged lymph nodes due to vaccination, the range of values was 5.5 to 0.4% at the large city and the rural health centre, respectively. The same was true for dizziness: significant differences between responses and expectations were confirmed (*p* < 0.01), with a range of 6.5 to 0.4% across the previously mentioned health centres. Taking into consideration convulsions, significant differentiation was confirmed (*p* < 0.05), and the highest incidence of this AEFI was confirmed in the small city health centre (3.2%), the lowest was the same as in the case of the majority of answers, which was observed in the rural health centre (0.4%). On the other hand, in cases of anaphylactic shock and other AEFI, the answers show no significant differences (*p* > 0.05).

**Table 3:** Occurrence of AEFI after vaccination in patients.

Item	Health centre			χ^2^ value	*p*-value*
	Large city (*n* = 310)	Small city (*n* = 372)	Village (*n* = 239)		
Have you experienced AEFI after vaccination? (df = 2)					
No	248 (80.0%)	305 (82.0%)	222 (92.9%)	18.981	<0.001
Yes	62 (20.0%)	67 (18.0%)	17 (7.1%)		
Fever (df = 2)					
No	260 (83.9%)	319 (85.8%)	226 (94.6%)	15.556	<0.001
Yes	50 (16.1%)	53 (14.2%)	13 (5.4%)		
Swelling at the vaccination site (df = 2)					
No	273 (88.1%)	342 (91.9%)	236 (98.7%)	22.111	<0.001
Yes	37 (11.9%)	30 (8.1%)	3 (1.3%)		
Enlarged lymph nodes (df = 2)					
No	293 (94.5%)	368 (98.9%)	238 (99.6%)	19.468	<0.001
Yes	17 (5.5%)	4 (1.1%)	1 (0.4%)		
Dizziness (df = 2)					
No	290 (93.5%)	360 (96.8%)	238 (99.6%)	14.450	0.001
Yes	20 (6.5%)	12 (3.2%)	1 (0.4%)		
Anaphylactic shock/ allergic reaction (df = 2)					
No	310 (100.0%)	371 (99.7%)	239 (100.0%)	1.477	0.478
Yes	0 (0.0%)	1 (0.3%)	0 (0.0%)		
Convulsions (df = 2)					
No	305 (98.4%)	360 (96.8%)	238 (99.6%)	6.269	0.044
Yes	5 (1.6%)	12 (3.2%)	1 (0.4%)		
Others (df = 2)					
No	305 (98.4%)	366 (98.4%)	234 (97.9%)	0.238	0.888
Yes	5 (1.6%)	6 (1.6%)	5 (2.1%)		

*p-value statistically significant at *p* < 0.05.

### 3.5 Self-reported AEFI among participants

Table 4 presents the results of AEFI incidence in the study’s family and close relatives. In contrast to AEFI in patients (Table 3), a higher percentage of individuals with post-vaccination complications was found, ranging from 32.3 to 34.4% in urban health centres and 8.8% in rural health centres. In cases of fever-conducted tests, a significant difference between observed and expected values (*p* < 0.01) was confirmed, with ranges of 26.6 to 6.7% at the small city health centre and the village health centre, respectively. During analysis of swelling data at the vaccination site, a significant difference (*p* < 0.01) was observed, with frequencies ranging from 16.8 to 2.1% at the large city health centre and the rural health centre, respectively. Considering the obtained frequencies in cases of enlarged lymph nodes, a significant difference (*p* < 0.05) between observed and expected values was obtained, with the highest frequency in the large city health centre (4.5%) and the lowest in the village health centre (0.4%). Pearson’s chi-square test also confirmed a significant difference (observed/expected values) for the dizziness response (*p* < 0.01), with the highest incidence of this AEFI noted in the large city health centre (10.3%) and the lowest in the village health centre (2.1%). Significant differences between health centres were also observed for convulsions (*p* < 0.01), with the highest incidence rate in urban health centres (about 5.2%) and the lowest in rural health centres (0.4%). No significant difference between observed and expected values was noted in the case of anaphylactic shock or allergic reaction (*p* > 0.05). Patients at the rural health centre had a lower incidence of post-vaccination complications than those in urban centres. Additionally, the health centre in a large city had a higher proportion of patients with AEFI than the health centre in the rural area (*p* < 0.01).

**Table 4:** Occurrence of AEFI after vaccination in family and close relatives of patients.

Item	Health centre			χ^2^ value	*p*-value*
	Large city (*n* = 310)	Small city (*n* = 372)	Village (*n* = 239)		
Is anyone close to you experienced AEFI after vaccination? (df = 2)					
No	210 (67.7%)	244 (65.6%)	218 (91.2%)	54.886	<0.001
Yes	100 (32.3%)	128 (34.4%)	21 (8.8%)		
Fever (df = 2)					
No	240 (77.4%)	273 (73.4%)	223 (93.3%)	37.775	<0.001
Yes	70 (22.6%)	99 (26.6%)	16 (6.7%)		
Swelling at the vaccination site (df = 2)					
No	258 (83.2%)	323 (86.8%)	234 (97.9%)	30.258	<0.001
Yes	52 (16.8%)	49 (13.2%)	5 (2.1%)		
Enlarged lymph nodes (df = 2)					
No	296 (95.5%)	360 (96.8%)	238 (99.6%)	8.153	0.017
Yes	14 (4.5%)	12 (3.2%)	1 (0.4%)		
Dizziness (df = 2)					
No	278 (89.7%)	341 (91.7%)	234 (97.9%)	14.193	0.001
Yes	32 (10.3%)	31 (8.3%)	5 (2.1%)		
Anaphylactic shock/ allergic reaction (df = 2)					
No	306 (98.7%)	365 (98.1%)	236 (98.7%)	0.546	0.761
Yes	4 (1.3%)	7 (1.9%)	3 (1.3%)		
Convulsions (df = 2)					
No	294 (94.8%)	353 (94.9%)	238 (99.6%)	10.470	0.005
Yes	16 (5.2%)	19 (5.1%)	1 (0.4%)		
Others (df = 2)					
No	283 (91.3%)	359 (96.5%)	238 (99.6%)	23.155	<0.001
Yes	27 (8.7%)	13 (3.5%)	1 (0.4%)		

^*^p-value statistically significant at *p* < 0.05.

### 3.6 Social influence, policy appraisal, and attitudes toward mandatory vaccination

Table 5 examines the potential influence of close relatives on the decision to vaccinate and on the assessment of vaccination policy in Poland. A comparison of the influence of close relatives on the vaccination decision, on a five-point scale, indicated statistically significant differences (*p* < 0.01). In large cities, 42.6 and 31.3% of patients chose the most frequent answers (respectively for sometimes and rarely) to the question ‘was the decision influenced by others (family, friends, or social groups)?’. At the small city health centre, the two most frequent responses were ‘never’ (33.3%) and ‘sometimes’ (29.6%). In the rural health centre, patients sometimes (53.6%) and often (33.9%) emphasized answers. In the second question, significant differences in responses (*p* < 0.05) were also observed regarding the assessment of the country’s vaccination policy. In large and small cities, the most frequent answer was ‘on average’ (50.6 and 53.8%, respectively), while at the rural health centre, the answer was ‘good’ (53.1%).

**Table 5:** Decisions about vaccinations, policy, and the mandatory nature of it.

Item	Health centre			χ^2^ value	*p*-value*
	Large city (*n* = 310)	Small city (*n* = 372)	Village (*n* = 239)		
Was the decision to vaccinate influenced to any extent by others (family, friends, social groups)? (df = 8)					
Never	44 (14.2%)	124 (33.3%)	3 (1.3%)	186.94	<0.001
Rarely	97 (31.3%)	84 (22.6%)	26 (10.9%)		
Sometimes	132 (42.6%)	110 (29.6%)	128 (53.6%)		
Often	31 (10.0%)	47 (12.6%)	81 (33.9%)		
Always	6 (1.9%)	7 (1.9%)	1 (0.4%)		
How do you assess the vaccination policy in the country? (df = 8)					
Very bad	8 (2.6%)	7 (1.9%)	2 (0.8%)	50.386	<0.001
Bad	36 (11.6%)	41 (11.0%)	5 (2.1%)		
On average	157 (50.6%)	200 (53.8%)	102 (42.7%)		
Good	102 (32.9%)	110 (29.6%)	127 (53.1%)		
Very good	7 (2.3%)	14 (3.8%)	3 (1.3%)		
Should vaccinations be mandatory? (df = 4)					
No	69 (22.3%)	88 (23.7%)	25 (10.5%)	80.814	<0.001
Yes	146 (47.1%)	160 (43.0%)	187 (78.2%)		
I do not have opinion	95 (30.6%)	124 (33.3%)	27 (11.3%)		

^*^p-value statistically significant at *p* < 0.05.

From the point of view, the question ‘if vaccinations should be mandatory?’ was also differentiated (*p* < 0.01); the most frequent answer was Yes, from 43.0% in a small city to 78.2% in the rural health centre. Additionally, in large and small city health centres, 22.3 and 23.7% of patients, respectively, chose the answer ‘no’. Additionally, the proportion of patients in cities who do not have an opinion on this question has increased from 30.6 to 33.3%.

### 3.7 Clinical predictors of vaccination uptake

Table 6 presents data on patients’ willingness to be vaccinated across all centres, depending on their previous illnesses and prior cardiovascular, respiratory, and metabolic diseases. The table lists vaccinations for which a logistic regression model could be built using the aforementioned independent variables. For other diseases, logistic regression models could not be created due to the low vaccination rate.

**Table 6:** Logistic regression model describing the willingness of vaccinated individuals against the most common diseases among adults and children.

Item	Disease		
	Influenza	COVID-19	Pertussis
Logistic regression model (*n* = 1,180)			
Vaccinated patients (*n*)	238	231	97
Non-vaccinated patients (*n*)	942	949	1,083
LR χ2 value	167.62	53.640	42.178
LR *p*-value*	<0.001	<0.001	<0.001
R2NK	0.693	0.645	0.601
AIC	964.44	1108.98	279.32
BIC	994.88	1139.42	309.76
AUC	0.72	0.64	0.63
Constituents of equation			
Intercept			
*p*-value	<0.001	<0.001	<0.001
−95% CI	2.043	−1.904	−2.398
+95% CI	1.682	−1.540	−1.954
OR	0.151	0.179	0.113
OR, −95% CI	0.124	0.149	0.091
OR, +95% CI	0.182	0.214	0.142
Prior illness			
*p*-value	<0.001	<0.001	0.997
−95% CI	2.051	0.923	−0.083
+95% CI	2.825	1.656	0.025
OR	11.037	3.630	0.000
OR, −95% CI	7.410	2.517	0.000
OR, +95% CI	16.441	5.237	0.000
Circulatory system diseases			
*p*-value	0.422	0.587	0.005
−95% CI	−0.076	−0.086	−2.918
+95% CI	0.032	0.049	−0.529
OR	0.978	0.982	0.178
OR, −95% CI	0.927	0.918	0.054
OR, +95% CI	1.032	1.050	0.589
Respiratory diseases			
*p*-value	0.001	0.010	0.001
−95% CI	0.587	0.243	0.667
+95% CI	2.156	1.807	2.701
OR	3.869	2.787	5.387
OR, −95% CI	1.764	1.275	1.949
OR, +95% CI	8.489	6.089	14.894
Metabolic diseases			
*p*-value	0.252	0.125	0.006
−95% CI	−0.166	−0.081	−2.820
+95% CI	0.633	0.065	−0.468
OR	1.263	1.339	0.193
OR, −95% CI	0.847	0.922	0.060
OR, +95% CI	1.883	1.944	0.626

*p-value statistically significant at *p* < 0.05.

All three logistic regression models were statistically significant according to the likelihood ratio χ^2^ test (*p* < 0.05). Nagelkerke’s R^2^ ranged from 0.601 for pertussis to 0.693 for influenza, indicating that the models explained a substantial proportion of the variability in vaccination status. The lowest AIC and BIC values were observed for the pertussis model (279.32 and 309.76, respectively), whereas the highest values were observed for the COVID-19 model (1108.98 and 1139.42, respectively); however, comparisons of these criteria across models with different dependent variables should be interpreted with caution. The influenza model demonstrated acceptable discriminatory performance (AUC = 0.72), while the COVID-19 and pertussis models showed poor discrimination (AUC = 0.64 and 0.63, respectively). VIF values ranged from 1.01 to 1.16 across all models, indicating no meaningful multicollinearity among the predictors. The maximum inflation of the standard error of a regression coefficient was approximately 7.7%.

For the decision regarding influenza vaccination, based on data collected from patients (*n* = 1,180), a significant (*p* < 0.01) effect of the independent variables on the dichotomous vaccination decision variable was found. In this case, a previous history of influenza and other respiratory diseases had a significant impact on the decision (*p* < 0.01, OR, respectively, 11.037 and 3.869). However, no significant effect (*p* > 0.05) of prior cardiovascular (OR = 1.548) or metabolic disease (OR = 2.441) was found in the logistic regression model (*p* > 0.05). Analyzing the logistic regression model for COVID-19 vaccination (*n* = 1,180), a significant effect of independent variables on the dichotomous variable was also found (*p* < 0.01). Similar to influenza, significant effects (*p* < 0.01) were observed for prior COVID-19 (OR = 3.630) and other respiratory illnesses individually (OR = 2.787). However, no significant impact (*p* > 0.05) was observed in the logistic regression model when prior circulatory disease (OR = 0.982) or metabolic disease (OR = 1.339) was considered. In the case of pertussis (*n* = 1,180), among the independent variables influencing the overall logistic regression model (*p* < 0.01), no significant effect of prior illness on vaccination was found (*p* > 0.05). However, prior respiratory (OR = 5.387), circulatory (OR = 0.178), and metabolic diseases (OR = 0.193) have a significant impact (*p* < 0.01) when considered individually in the equation.

## 4 Discussion

### 4.1 Principal findings in context

In this cross-sectional study conducted in three geographically distinct primary care settings in Poland, we identified pronounced urban–rural differences in vaccination uptake, vaccination-related attitudes, trust in the healthcare system, and self-reported adverse events following immunisation (AEFI). Vaccination coverage was highest in the rural centre and lower in urban centres, whereas AEFI reporting, both personal and reported in close relatives, was more frequent in urban populations. It must also be emphasized that the observed 100% vaccination rate in the rural health centre should be interpreted with caution, as it may reflect centre-specific recruitment characteristics resulting from the project-specific procurement procedure rather than the underlying vaccination coverage of the wider rural population. Therefore, these findings should be regarded as health centre context-specific observations and represent a limitation of the study.

In multivariable models, respiratory comorbidity and prior infectious disease experience were independently associated with vaccination uptake, particularly for influenza and COVID-19.

These findings are consistent with the broader literature, which demonstrates that vaccine uptake and hesitancy are shaped by multilevel determinants that operate at the individual, interpersonal, and structural levels (1, 3, 4, 8). Larson et al. (1) highlight that vaccine confidence is not only a function of personal belief but also of trust in institutions and information ecosystems. Similarly, Dubé et al. (3) emphasize that hesitancy reflects a dynamic interaction between perceived risks, benefits, and contextual influences. Our results extend these frameworks by demonstrating how such determinants manifest within a single national healthcare system across distinct geographic environments.

### 4.2 Geographic gradients: information environments and institutional trust

The observed differences in trust and policy assessment between rural and urban participants align with research indicating that institutional confidence is central to vaccine behavior (1, 4). Wiysonge et al. (4) describe vaccine hesitancy during the COVID-19 era as deeply embedded in broader trust landscapes shaped by political discourse and digital information flows. In our study, rural respondents reported higher levels of trust in the healthcare system and more favorable views of vaccination policy, patterns consistent with research suggesting that communities characterized by closer physician–patient continuity may demonstrate greater acceptance of preventive interventions (5, 6, 10).

Smith et al. (6), analyzing rural–urban disparities in the United States, report that although rural regions may face access barriers, community cohesion and local trust can buffer hesitancy. Our findings suggest a similar mechanism in the Polish context. Conversely, urban participants reported lower trust and higher AEFI reporting, which may reflect greater exposure to heterogeneous and sometimes conflicting information sources. Betsch et al. (2) identify exposure to misinformation and risk amplification as key psychological antecedents of vaccine hesitancy, particularly in digitally saturated environments. Karafillakis and Larson (8) further demonstrate that perceived vaccine risks in European populations often exceed objective risk levels, particularly when narratives about safety circulate through media and peer networks.

Urban populations may therefore experience both informational abundance and interpretive uncertainty. The coexistence of high service availability with lower trust underscores that structural access alone does not guarantee uptake. Instead, informational coherence and credibility appear equally important (1, 4, 14). Our findings suggest that access to vaccination-related information, the perceived quality of that information, and trust in healthcare institutions are important contextual determinants of vaccination behavior. Individuals who perceive information as accessible, reliable, and consistent may be more likely to develop confidence in vaccination recommendations and accept preventive interventions. Similar observations have been reported among older adults. Gürel Köksal et al. demonstrated that vaccination status for influenza and pneumococcal diseases is associated with a range of individual and contextual factors. At the same time, Odziemczyk-Stawarz and Perek-Białas highlighted the importance of socio-cultural and psychosocial determinants, including perceptions, trust, and access to information, in shaping vaccination decisions among older Europeans. Our results support these observations and suggest that strengthening trust in primary healthcare and improving the accessibility and quality of vaccination information may contribute to more favorable vaccination attitudes and potentially higher vaccine uptake (21, 22).

### 4.3 Urban–rural patterns in AEFI reporting

Self-reported AEFI were least frequent in the rural centre and most frequent in the large-city centre. It is important to emphasize that the AEFIs in this study were self-reported rather than clinically adjudicated. The literature indicates that perceptions and reporting of AEFI are influenced by expectations, symptom attribution, and media framing (3, 8). Guo et al. (12) demonstrate that vaccine attitudes are shaped not only by objective health events but also by their interpretation within social and informational contexts. Higher reporting in urban settings may therefore represent increased symptom vigilance rather than biological differences in reactogenicity.

Notably, AEFI reported in close relatives exceeded personal AEFI reporting. This aligns with evidence that vicarious experiences play a decisive role in shaping vaccine attitudes (4, 13, 17, 23, 24). Babicki and Mastalerz-Migas (16) observed that peer narratives and household-level discussions significantly influenced perceptions of the COVID-19 vaccine in Poland. Indirect exposure to adverse event narratives can amplify perceived risk, even in the absence of personal experience, thereby influencing behavioral intentions (4, 17).

Our findings reinforce the need to consider not only individual-level experiences but also the diffusion of safety perceptions within family and social networks.

### 4.4 Comorbidity, vulnerability perception, and behavioral response

Respiratory comorbidity was consistently associated with higher vaccination uptake across models. This association is biologically and behaviorally plausible. Sheinfeld Gorin (11) and Guo et al. (12) highlight that perceived susceptibility and perceived severity are central drivers of vaccine acceptance. Individuals with respiratory conditions may more readily internalize their vulnerability to influenza and COVID-19, translating clinical risk into preventive behavior.

By contrast, circulatory and metabolic conditions were not significant predictors in most models. This finding parallels mixed evidence in the literature. While some studies suggest higher uptake among patients with chronic disease due to medical recommendations (11, 13), others demonstrate persistent safety concerns and ambivalence among individuals with cardiometabolic disorders (3, 8, 13). Cardiovascular and metabolic risks may be less intuitively linked to vaccine-preventable infections than to respiratory illness. This discrepancy between clinical indication and perceived risk underscores the importance of targeted risk communication within primary care.

Prior experience with infectious diseases was a strong predictor of influenza and COVID-19 vaccination. Such findings support behavioral models emphasizing experiential learning. Direct disease experience appears to increase salience and perceived benefit of vaccination (12–14), reinforcing prevention-oriented behavior.

### 4.5 Comparison with national and international evidence

The geographic heterogeneity observed in this study mirrors prior Polish data documenting variability in COVID-19 vaccine acceptance across regions and sociodemographic groups (10, 13, 16, 19, 20). Kurpas et al. (10) describe vaccine hesitancy in Central and Eastern Europe as influenced by sociocultural and digital environments, including historical legacies of institutional distrust. Sobierajski et al. (13) further report pandemic-era shifts in attitudes linked to policy communication and political framing.

Internationally, similar urban–rural gradients have been documented (6, 9, 17). Lo Moro et al. (9) observed differential acceptance across Italian regions, while other studies emphasize the interplay between misinformation exposure, risk perception, and trust (1–3, 8). Taken together, these findings situate our results within a broader European and global context in which the interaction of clinical vulnerability, information ecology, and institutional credibility shapes vaccination behavior.

## 5 Conclusion

This cross-sectional primary care study demonstrates that vaccination uptake, vaccination attitudes, trust in the healthcare system, and self-reported adverse events following immunisation differ substantially across urban and rural contexts within a single national healthcare framework. Rural participants exhibited higher vaccination coverage and lower reporting of AEFI, alongside higher levels of trust in healthcare institutions and more favorable assessments of vaccination policy. In contrast, urban populations demonstrated lower coverage and more frequent reporting of AEFIs, suggesting that informational environments and institutional confidence may shape both behavioral uptake and symptom attribution. Respiratory comorbidity and prior experience with infectious disease emerged as consistent independent predictors of vaccination, particularly for influenza and COVID-19. In contrast, circulatory and metabolic conditions were not reliably associated with uptake, indicating a potential gap between objective clinical risk and perceived vulnerability.

These findings underscore that vaccination behavior cannot be understood solely at the individual level. Geographic context, clinical vulnerability, and the surrounding information ecosystem jointly influence both decision-making and safety perceptions. Primary care remains a critical setting for addressing these gradients through diagnosis-linked counseling, context-sensitive communication strategies, and structured discussions about expected adverse events. Future research should incorporate longitudinal designs, multilevel modeling, and measurement of health literacy and media exposure to further clarify the pathways linking geographic context, risk perception, and vaccination behavior.

### 5.1 Strengths and limitations

#### 5.1.1 Strengths

This study has several methodological strengths. It integrates clinical and survey data within real-world primary care settings, enhancing ecological validity and practical relevance. Linking self-reported responses to medical records reduces misclassification of vaccination status and comorbidities, thereby strengthening internal validity. Inclusion of three geographically and socially distinct settings within the same healthcare system permits controlled contextual comparison while limiting systemic heterogeneity. The analytic approach included multivariable logistic regression to identify independent associations between comorbidity profiles, prior infection, and vaccine uptake, extending beyond descriptive comparisons. Fifth, the assessment of both personal and vicarious AEFI experiences provides a novel dimension rarely incorporated in primary care vaccine studies.

#### 5.1.2 Limitations

Several limitations must be acknowledged. The cross-sectional design precludes causal inference. Participating centres were recruited through a project-specific procurement procedure and therefore do not constitute a nationally representative sample of Polish primary care institutions. Consequently, the findings should be interpreted as context-specific observations rather than national estimates. This limitation is particularly relevant for the rural health centre, where all recruited participants were vaccinated. Consequently, the predictive performance of the regression models may have been affected by the limited variability in vaccination status, and the findings should be interpreted with caution when extrapolated to broader geographical settings.

Participation was limited to individuals attending participating centres and consenting to data linkage, potentially introducing selection bias and over representation of individuals more engaged with healthcare services. AEFI and attitudinal measures were self-reported and subject to recall and attribution biases, as well as differential reporting norms. AEFI in relatives was second-hand reports and not clinically verified. Differences in reporting reflect perception rather than physiological variation. Relevant determinants, such as health literacy, digital media exposure, and political orientation, were not measured, consistent with prior work that identified these factors as significant contributors to vaccine confidence (2, 14). High non-response rates for income may limit socioeconomic adjustment. The analysis did not incorporate multilevel modeling to account for clustering by centre, which could influence variance estimates. Additionally, regression models were limited to vaccines with sufficient uptake, constraining the generalisability of modeling results.

## Data Availability

The original contributions presented in the study are included in the article/supplementary material, further inquiries can be directed to the corresponding author.
